# Simulating Microdosimetry in a Virtual Hepatic Lobule

**DOI:** 10.1371/journal.pcbi.1000756

**Published:** 2010-04-22

**Authors:** John Wambaugh, Imran Shah

**Affiliations:** National Center for Computational Toxicology, Office of Research and Development, U.S. Environmental Protection Agency, Research Triangle Park, North Carolina, United States of America; Medical College of Wisconsin, United States of America

## Abstract

The liver plays a key role in removing harmful chemicals from the body and is therefore often the first tissue to suffer potentially adverse consequences. To protect public health it is necessary to quantitatively estimate the risk of long-term low dose exposure to environmental pollutants. Animal testing is the primary tool for extrapolating human risk but it is fraught with uncertainty, necessitating novel alternative approaches. Our goal is to integrate *in vitro* liver experiments with agent-based cellular models to simulate a spatially extended hepatic lobule. Here we describe a graphical model of the sinusoidal network that efficiently simulates portal to centrilobular mass transfer in the hepatic lobule. We analyzed the effects of vascular topology and metabolism on the cell-level distribution following oral exposure to chemicals. The spatial distribution of metabolically inactive chemicals was similar across different vascular networks and a baseline well-mixed compartment. When chemicals were rapidly metabolized, concentration heterogeneity of the parent compound increased across the vascular network. As a result, our spatially extended lobule generated greater variability in dose-dependent cellular responses, in this case apoptosis, than were observed in the classical well-mixed liver or in a parallel tubes model. The mass-balanced graphical approach to modeling the hepatic lobule is computationally efficient for simulating long-term exposure, modular for incorporating complex cellular interactions, and flexible for dealing with evolving tissues.

## Author Summary

Virtual tissues are emerging as a powerful tool for computational biology. By encoding known biology into a simulation of tissue function, gaps in knowledge can be identified. As a simulation of tissue function, *in silico* experiments can be performed inexpensively and rapidly. There are over 6000 chemicals produced in large quantities that may be present in our environment, many of which have not been thoroughly examined for human toxicity. Traditional toxicity testing is expensive, lengthy, and relies heavily upon the use of animals. For this reason *in vitro* toxicity testing techniques are being developed. However, techniques are needed to relate *in vitro* results to *in vivo* conditions. The liver is often the first tissue to show signs of toxicity and therefore a predictive liver toxicity simulator would be a powerful tool to reduce the financial and animal cost of toxicity testing. As a first step, we have developed a model for relating environmental exposure to cell-level concentrations; a model for virtual tissue microdosimetry. We identify regimes in which this approach is equivalent to previous techniques, as well as regimes where large cell-to-cell variability exists. This variability should have consequences both for normal liver function and the onset of injury.

## Introduction

As the number of man-made environmental chemicals continues to grow, there is an urgent need to develop effective tools to test their potential risk to humans. The number of environmental chemicals that are produced in substantial quantities now numbers approximately 10,000 [1]. In order to determine the potential risk to humans of exposure to these compounds, it is critical to establish a *dose-response* curve – the functional dependence of toxic endpoints, *e.g.* hepatic lesions, on exposure to that compound. Traditional long-term animal testing to determine dose-response is time consuming, expensive, and requires the sacrifice of thousands of animals without clear relevance to humans. Recognizing this need for new approaches to toxicity testing [2], [3], [4], the U.S. Environmental Protection Agency is conducting ongoing efforts to collect *in vitro* data [5] to make inferences about *in vivo* toxicity in both test animals [6] and humans [7].

Without appropriate context, *in vitro* testing is insufficient for predicting effects *in vivo*. Context can be established through informatics, *i.e.* correlating *in vitro* data with known *in vivo* phenotypes, or modeling efforts in which abstract rules are hypothesized to determine *in vivo* outcomes as a function of variables, some of which may be determined *in vitro*. Whereas empirical models describe the available data and are therefore best limited to interpolation, physiologic models attempt to describe the underlying biology in sufficient detail to emulate the true dynamics. Physiologic models generate new hypotheses which can subsequently be tested to refine the model. Both informatics and modeling approaches create frameworks without which there could be little meaningful interpretation of *in vitro* data.

**Figure 1:**
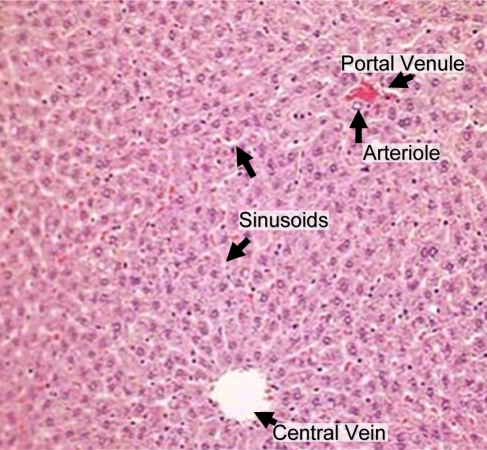
Hepatic lobules receive nutrient-rich blood from the gut through the portal venule and oxygen-rich blood from the lungs through arterioles. Blood flows past sheets of hepatocytes through the sinusoids and into the central vein. Image adapted from an original by Amber Goetz, first published in [42].

Our goal is to establish an *in silico* model for dose-response that can be calibrated using *in vitro* characterizations of chemical effects. The liver is often the site of initial exposure to hazardous compounds and their metabolites due to first-pass metabolism of blood from the gastro-intestinal tract via the hepatic vein. In mammals the hierarchical structure of the liver terminates in 10^5^ to 10^6^ functional units called lobules [8] first identified by Kiernan [9]. Each hepatic lobule receives blood from up to six portal triads, each typically consisting of a hepatic arteriole and a portal venule in addition to a bile ductule [10]. Blood flows through intervening spaces between the cells, *i.e.* sinusoids [11], and drains into the central vein. Hepatocytes are arranged in plates one to two cells thick, organized radially around the central vein. A two-dimensional slice of a hepatic lobule is shown in Figure 1. Compounds within the blood are exchanged with the hepatocytes sequentially as blood passes through the sinusoids. The action of the enzymes within the hepatocytes on compounds produces metabolites that may be more or less harmful than the parent compound. Although mechanisms of chronic chemical-induced injury are not completely understood, it is believed to involve multiscale molecular and cellular interactions that culminate in tissue damage.

Tissue dosimetry is traditionally estimated using physiologically-based pharmacokinetic (PBPK) models. A PBPK model consists of a system of ordinary differential equations (ODEs) for the concentration of a compound (or compounds) in different tissues. Typically some key tissues are treated as separate compartments for which a tissue-specific concentration is calculated, while other tissues are modeled using aggregate compartments. More complicated dynamics within a tissue, such as diffusion or membrane transport, are often modeled with additional sub-compartments but each sub-compartment is well-mixed. The equations are parameterized by subject- or species-specific physiologic parameters such as cardiac output and tissue volumes as well as compound-specific parameters such as diffusion/transport rates and tissue-specific plasma to tissue “partition coefficients” corresponding to the assumption of a rapidly-established equilibrium between concentration of compound stored in the tissue and the concentration of compound in the plasma flowing through the tissue. PBPK models relate the concentration of compounds inhaled or ingested from the environment to internal tissue doses [12], [13], [14].

In addition to the well-mixed approach, the parallel tubes model of liver function has often been used to calculate *in vivo* hepatic metabolism based upon *in vitro* measures such as intrinsic hepatic clearance [15], [16]. Typically used at steady-state, the parallel tubes model assumes that each lobule is a tube connecting a portal triad and central vein, along which concentration varies spatially.

Though *in vitro* studies typically average over the response of a many hepatocytes within a well, hepatocyte function is known to vary significantly *in vivo* [17], *e.g.*, hepatocytes near the central vein may express very different levels of enzymes than those nearer to the portal triad. For this reason the lobule is divided into zones of approximately similar hepatocyte function depending on location within the lobule. The heterogeneity between these zones is thought to arise from gradients in oxygen availability, exposure to nutrients from the portal venules, and hormone concentration [18]. Modeling the differences between regions of the lobule should provide key insights into the differences between phenomena observed in homogenous *in vitro* conditions and heterogeneous *in vivo* reality.

The first multi-compartment geometric model of the liver was developed by Andersen et al. [19]. In that model there were no cells, but the concentrations of compounds in different zones of lobules were modeled continuously and could therefore be coupled to a PBPK model. Liu et al. [20] have followed a similar sub-compartment coupled to PBPK approach for modeling zonal heterogeneity due to transporters and enzymes. Recent approaches to simulating the response of the liver include that of Ohno et al. [21] who coupled independent realizations of a model for cellular dynamics into a linear array to allow some instances of the model to be close to the source of nutrients and foreign compounds while others were further removed. Höhme et al. [22] have developed a discrete model of the hepatic lobule that considers the biochemical forces between hepatocytes to simulate recovery following acute chemical toxicity.

Ierapetritou et al. [18] recently conducted a thorough review of liver tissue simulation approaches in which they summarize the previously mentioned approaches as well as higher dimensional models including fluid dynamics approaches based upon approximations of the Navier-Stokes partial differential equations. Such approaches are data- and computationally-intensive, especially given the convoluted and dynamic cellular boundary of the sinusoidal spaces.

Hunt et al. [23] have taken a unique agent-based approach with individual hepatocytes represented by agents wherein metabolism can occur. The environment of the agents is determined using a hybrid graph and grid approach in which compounds are represented by objects moving through the lobule. Cell-oriented agent-based modeling (ABM) of tissues offers a number of unique advantages [24], [25]. First, since cells are the functional units of tissues, the ABM has more physiologic relevance than a continuum model. Second, the agent responses can be calibrated and verified through comparison with actual cells *in vitro* (or *ex vivo*). Third, spatial outcomes from the ABM can be translated to histopathologic effects such as acute lesions and tumor formation. While the agent-based strategy is suitable for modeling tissue responses, the approaches to the liver taken so far have not provided a framework for estimating tissue dosimetry. Though the spatial distribution of a compound has previously been modeled, past approaches have represented compounds as agents that are difficult to link to traditional exposure modeling. Due to the spatial heterogeneity of the hepatic lobule, both molecular and cellular, it is important to model the microanatomic distribution of chemicals and to relate this to continuous variation in chemical concentration resulting from changes in human environmental exposure.

We have implemented a microdosimetry model that relates whole-body chemical exposures to cell-scale concentrations. Our objective was to develop the framework for simulating the microanatomic distribution of various environmental chemicals in a canonical lobule for extended periods of time ranging from hours to months. This required an approach that is quantitative, efficient in computational resources, and sufficiently flexible to account for anatomic changes (due to chemical insult or other factors) [26]. First, we approximated the microanatomic architecture of the hepatic vasculature and parenchyma assuming a discrete topology by a graphical model. This allowed us to systematically explore the consequences of morphologic changes on the concentration distribution. Second, we transformed the sinusoidal elements of the vascular network into a system of microscopic well-mixed compartments through which material flow was assumed to be one-dimensional. Third, we connected the virtual lobule to a PBPK model to relate individual exposure to microdosimetry. For a range of physiologically relevant morphologic parameters we evaluated the microdosimetry in response to xenobiotic exposure levels and varying physico-chemical attributes.

## Results

### Discrete Graphical Model of Sinusoidal Network

The two dimensional morphologic characteristics of the mammalian hepatic lobule were represented as a discrete connectivity graph, in which the edges captured spatial proximity. The two main anatomic entities considered are hepatocytes, the parenchymal cells responsible for the metabolism of chemicals, and vasculature, *i.e.* sinusoids through which chemicals flow to the hepatocytes. These are represented by different node types including: hepatocytes, sinusoidal primitives, arterial and venous sources, and the central vein, while edges represent connectivity and spatial proximity between the nodes. Mass transfer through the sinusoidal network occurs through edges: The edges connecting vascular nodes transfer material through the sinusoids, whereas edges between the vascular and cellular nodes exchange material between the sinusoids and parenchyma.

**Figure 2:**
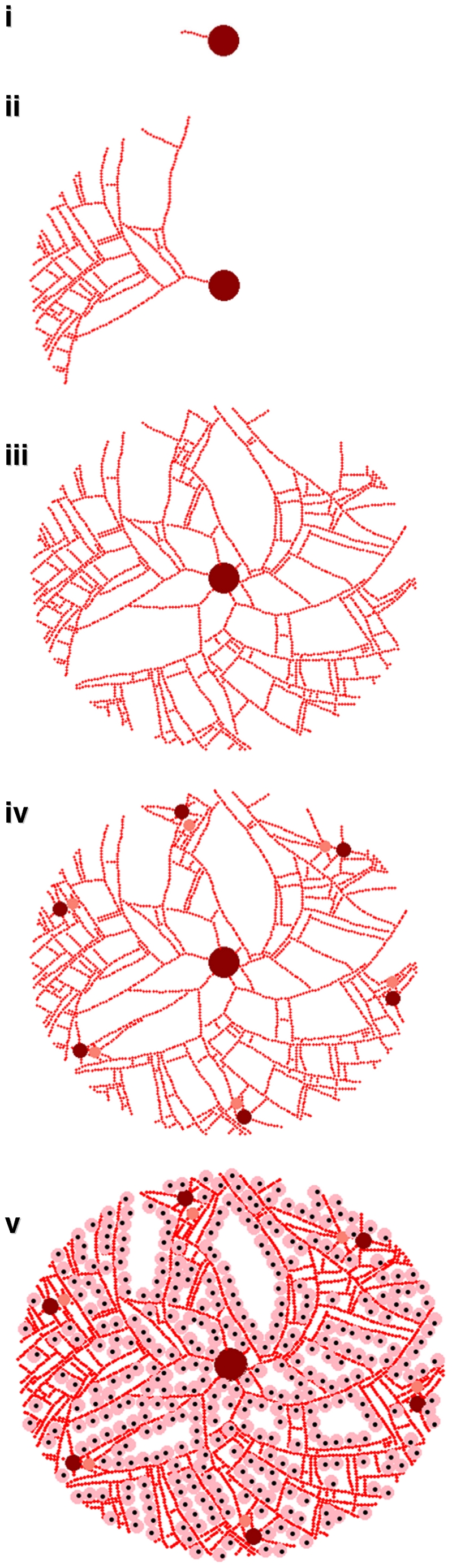
The virtual lobule morphology is constructed iteratively. First, sinusoids outward from the central vein (i). In addition to small random variations in the direction of propagation, the sinusoids branch into two sinusoids pointed away from the central vein with probability P_br_ (ii). Multiple sinusoids are started from the central vein in an attempt to fill space (iii). Portal “triads” consisting of arterioles and venules through which blood enters the lobule are added to the perimeter of the lobule and connected to the vasculature (iv). Finally, the sinusoids are lined with hepatocytes as space allows (v).

A simplified geometry of the lobule was defined using the following morphologic parameters: the number portal triads (defining the vascular inputs), the branching factor of the sinusoids, the number of sinusoids entering the central vein, and the sizes of sinusoids, hepatocytes, and the lobule. The graphical model of the lobule was constructed algorithmically using these parameters and visualized spatially (Figure 2). The “virtual lobules” generated in this manner presented a complex sinusoidal architecture representing a substantial challenge for estimating the distribution of xenobiotics and nutrients.

The graphical model of the lobule was generated iteratively (the algorithm is described in the methods section). The sinusoidal network was constructed starting with the central vein and extending radially outwards to the portal region. Beginning with a node representing the central vein, sinusoid primitives (nodes) were sequentially appended to form the initial vasculature. Small random variations in the placement and branching of sinusoidal primitives were used to reconstruct the histologic appearance of a hepatic lobule. Second, the hepatic arterioles and portal venules, were placed at the perimeter of the lobule and connected sinusoidal network. Third, the parenchymal cells were placed contiguously with the sinusoidal network. Because we chose to connect the portal venule and arterioles to the central vein in two dimensions the spatial layout was not completely space-filling.

**Figure 3:**
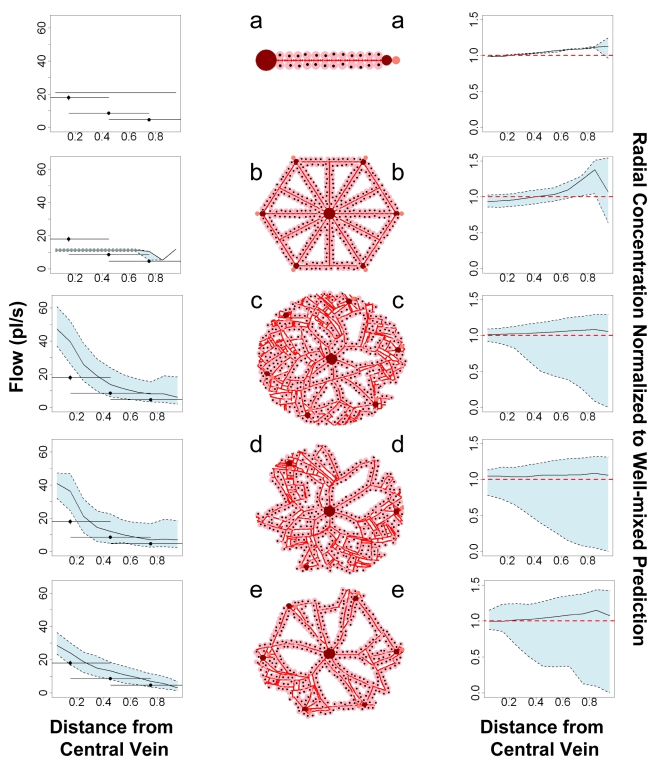
Five different lobule morphologies were examined. They are: a) one portal triad, no branching or noise, b) six portal triads with noise and additional sinusoids, c) six portal triads, 10% chance of branching, d) three portal triads with 10% branching, and e) six portal triads with 5% chance of branching. Though the overall layout (middle column) can be compared qualitatively with physiology, we evaluate these geometries by comparing the flow (left-hand column) predicted for a rat with *in vivo* measurements of flow in rat sinusoids (Komatsu et al. (1990) [31]). We also compare (right-hand column) the radial dependence of concentration at t_max_ with the prediction for a well-mixed compartment with equivalent metabolic clearance (heavy dashed line). Comparison of profiles b-e with profile a provides an approximate comparison to a parallel tubes prediction. The solid line indicates the mean for multiple lobules and sinusoids, while the shading indicates the 95% quantile (variability).

The approach described above is flexible, allowing the generation of diverse lobular topologies through which flows can be simulated. Five basic morphologies were examined, as depicted in Figure 3, in which the number of portal triads (more accurately *dyads* since bile was neglected), the probability of sinusoid branching *P_br_*, and the presence of random noise were all varied. No random noise or branching and one portal dyad produced a lobule with a single tube (panel a in Figure 3) that in the limit of many sinusoidal segments approaches a parallel tubes model. With multiple portal dyads a classical lobule structure [27] that allows both direct flow from the portal triads to the central vein and mixing flow between portal triads is produced (panel b in Figure 3). A 10% chance of sinusoid branching (panels c and d) produced nearly space-filling lobule graph while a 5% chance of sinusoid branching (panel e) did not.

Miller et al. (1979) observed that the branching of sinusoids is greater near the portal triad than near the central vein [28]. Human lobules have been observed to typically have between three and six portal triads per lobule [8], [10], [27], though many “triads” actually consist of dyads missing either an arteriole, bile duct, or most commonly a portal venule [10]. Given these observations, we believe that the geometries that include multiple portal triads and random branching of the sinusoids (panels c, d, and e) appear qualitatively more physiologic.

### Blood Flow in the Sinusoidal Network

**Figure 4:**
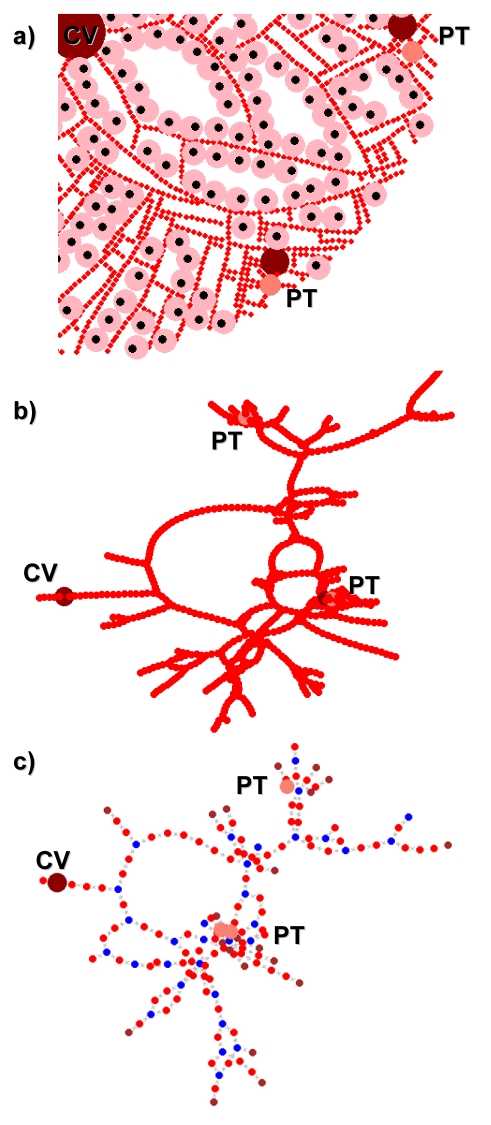
Sinusoid connectivity was represented with a graph. Spatial proximity between sinusoids within simulated lobule (a) was used to generate connectivity graphs (b), which are aggregated (c) in order to solve for flow from the portal triads to the central vein using ODEs.

**Figure 5:**
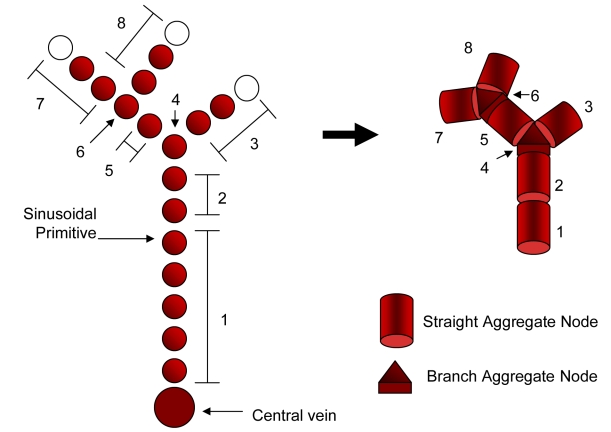
Similar nodes were aggregated to reduce the complexity of the sinusoid connectivity graph.

Blood circulation through the graphical model of the vasculature was simulated as a network flow (Figure 4). Because the sinusoidal diameter is much smaller than hepatocytes [29], there are a large number of sinusoid primitives in each virtual lobule. To efficiently solve for the flow, the sinusoid primitives were aggregated into the following components: “straight” or linear sequences and “branch” sections where straights meet and mix. As shown in Figure 5, graph aggregation results in a smaller graph that preserves the spatial distribution of the sinusoids. Each aggregated node was assumed to be well-mixed, that is, each constituent sinusoid primitive *i* has the same concentration *C* ^μ^_i_ (see Table 1 for a list of all symbols used in this document).

**Table 1:** List of Symbols.

Symbol	Definition
P_br_	Sinusoidal Branching Probability
*G(V,E)*	sinusoid graph consisting of vertices (nodes) V and edges E
*Q* ^μ^ *_ij_*	Micro flow rate (L/h) across from node *i* to node *j*
*F_i_*	Total flow into node *i*
Q^μ^_art_	Micro flow rate (L/h) through each arteriole
Q^μ^_art_	Micro flow rate (L/h) through each venule
R_liv∶lob_	Ratio of liver to lobule volume
Q_gut_	Flow rate (L/h) through gut tissue
Q_liv_	Flow rate (L/h) of arterial blood into liver
*C* ^μ^ *_i_*	Concentration of within aggregate sinusoid *i* and each constituent sinusoid
C_liv_	Concentration for a well-mixed liver compartment
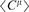	Concentration averaged over the lobule
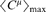	Maximum concentration averaged over the lobule
*C* ^μ^ *_i,max_*	Maximum concentration within aggregate sinusoid *i*
t_max_	Time at which maximum average concentration is reached

Mass-balanced flow through the aggregate graph was determined by solving for the flow across each edge of the sinusoid graph *G(V,E)* due to the sources at both the arterial and venous elements of each portal triad. In general, solving for network flow from node *i* to node *j* across edge E_ij_ requires *|E|* different weights *Q* ^μ^ *_ij_* (*i.e.*, flow rates). Mass-balance provides only *|V|* constraints – one at each node – so additional constraints were needed.

We made use of the hemodynamical equivalent of Ohm's law [30], [31]:
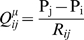
where P_i_ is the pressure at node *i* and the resistance 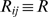 was assumed to have the same value R for all edges. We note that R_ij_ could be determined using schemes such as the cross-sectional area of each branch. Hemodynamics provides |E| additional constraints, but introduces |V| additional unknown pressures P_i_. Together with mass balance we have |E|+|V| constraints for |E|+|V| unknowns. This system of equations can be represented with a matrix and, given source flows and outlet pressure, can be solved by diagonalization. Since we are not currently interested in sinusoidal pressure, R and the outlet pressure were taken as one. This assumption does not effect the quantitative values of *Q* ^μ^ *_ij_* since they depend only on the relatively values of P_i_.

As can be seen in Figure 4, randomly generating sinusoids can lead to dead-end sinusoids for which no flow is predicted. These sinusoids are removed from the lobule and additional hepatocytes are added where possible.

To evaluate the appropriateness of these assumptions and the suitability of the approach to arbitrary graphical structures, we return to Figure 3, where predictions are made for a rat liver lobule and compared to measurements made by Komatsu et al. (1990) for the radial dependence of flow of erythrocytes in the sinusoids with distance from the central vein. *In vivo* microscopy was used by Komatsu et al. to observe the exposed livers of ten Sprague-Dawley rats and flow was measured in three zones – near the central vein, near the portal venule, and intermediate [31]. Flow was observed to increase with distance from the portal venule, presumably as blood from the portal arteriole and other portal triads mixed in. As can be seen on the left-hand side of Figure 3, only geometries where random branching is present (panels c, d, and e), produce profiles with increasing flow as the central vein is approached. Given the indeterminacy in where flow was measured relatively to the central vein, it is hard to determining the precise radial profile of the flow. All geometries produce mean flow within a factor of two of the measured values, supporting the approximate appropriateness of this graphical approach to hemodynamics in the hepatic sinusoids. A list of simulation parameters used is given in Table 2.

**Table 2:** Lobule Simulation Parameters.

Oral dose	10 µMol
Number of Lobules per Ensemble Analyzed	50
Agent-based model steps per Iteration	8
time per iteration	0.2 h
Total hours simulated	5
Number of Portal Triads	6
Number of Sinusoid starts at central vein	6
Sinusoidal Branching Probability *P_br_*	10%
Radius of Lobule	15 hepatocytes
diameter of hepatocyte	100 µm (assumed)
Thickness of lobule	23.5 µm [29]
Diameter of sinusoid primitive	25 µm [29]

### Chemical Distribution in the Sinusoidal Network and Cells

**Figure 6:**
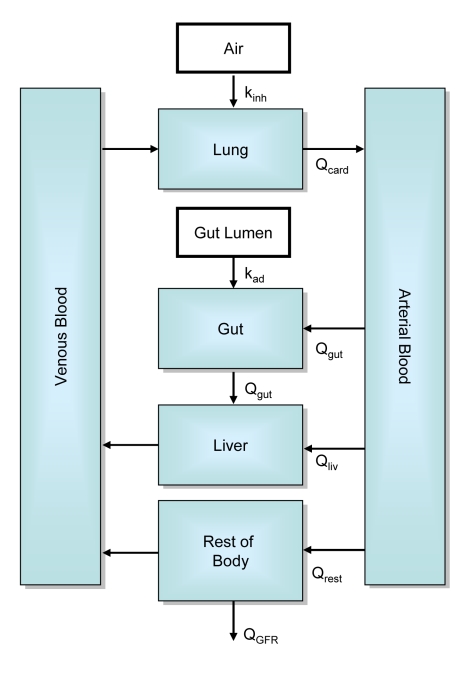
A physiologically-based pharmacokinetic model was used to relate oral and inhalation exposure to blood flow into the liver.

The final step needed to determine the concentration *C* ^μ^ *_i_* for each sinusoid *i* is to find the concentration of compound(s) in the blood flowing into the liver. Our approach requires the rate and chemical concentration(s) for blood flow from the gut and the hepatic arteries. We used a simple PBPK model (Figure 6) with oral and inhalation routes of exposure (PBPK model parameters are listed in Table 3). Microdosimetry for each lobule was determined from the pharmacokinetic exposure model by assuming an arteriole flow equal to Q^μ^_art_ = Q_liv_/R_liv∶lob_/N_PT_ and a venule flow Q^μ^_ven_ = Q_gut_/R_liv∶lob_/N_PT_ where R_liv∶lob_ is the ratio of liver to lobule volume and N_PT_ is the number of portal triads per central vein. Concentrations within the lobule are determined by solving
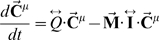
where *M_i_* is the summed clearance of all hepatocytes adjacent to aggregate sinusoid *i*, and **I** is the identity matrix. Note that at steady state the flow can be determined from just the geometry *G(V,E)* and the metabolism M, in which case 
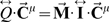
 and there is no need to solve for dynamic concentration changes, since new concentrations can be calculated analytically.

**Table 3:** Parameters Used for PBPK Model.

Parameter	Value	Source
Qcard	336 L/h	[43]
Qgut	66 L/h	[43]
Qliv	18 L/h	[43]
Qgfr	7.5 L/h	[43]
Qrest	252 L/h	[43]
Bodyweight	70 kg	assumed
Lean Fraction of BW	0.7	[19]
Vart, Vven	0.025 L/kg lean bw	[19]
Vgut	0.0165 L/kg bw	[19]
Vliv	0.035 L/kg lean bw	[19]
Vlung	0.27 L	[44]
Vrest	0.6 L/kg bw – (Vart+Vven+Vgut+Vliv+Vlung)	[43]
k_ad_, k_inh_, K_rest∶plas_, K_liv∶plas_, K_gut∶plas_, R_blood∶plas_, f	1	assumed

**Figure 7:**
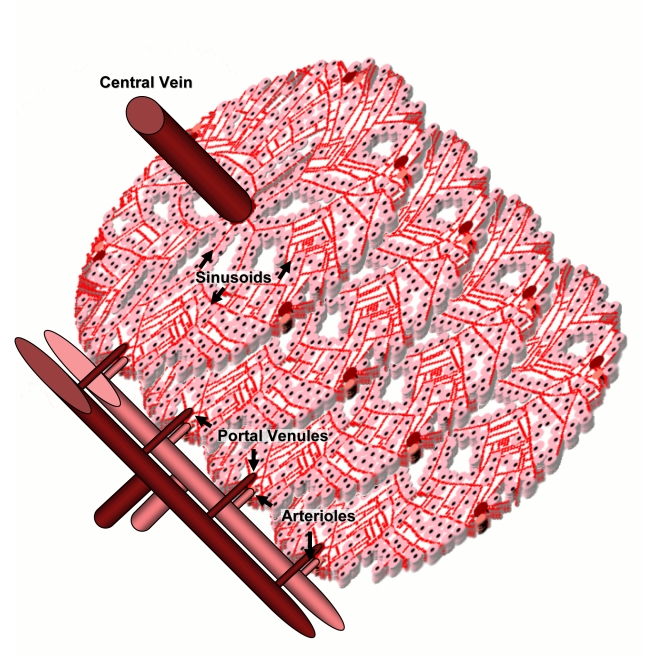
A physiologic lobule is a three-dimensional polyhedron with a volume between 0.1 and 0.9 µL [8]. Our (quasi-)two-dimensional simulated lobule is assumed to have a thickness equal to a sinusoidal diameter (23.5 µm [29]). Therefore many identical simulated lobules in parallel are needed to fill the volume of one physiologic lobule. Blood flow to the simulated lobules is divided by R_liv∶lob_, the ratio of the volume of the whole liver to the volume of single lobule.

For a completely physiologic, three-dimensional lobule R_liv∶lob_ would be equal to the number of lobules in the liver – approximately 10^6^ [32]. We determined R_liv∶lob_, the ratio of the total volume of the liver to the total volume of the sinusoidal spaces and hepatocytes in the simulated lobule, to be approximately 10^8^, which is roughly 100 times greater than the physiologic value. We expect a greater value for two reasons: First, many components of the lobule other than the sinusoidal spaces and hepatocytes, such as endothelial and stellate cells, extracellular space, and bile ducts, contribute to the volume of the lobule. Including these additional components, and therefore increasing the volume of the simulated lobule, will reduce R_liv∶lob_. Second, each simulated lobule is assumed to have a thickness equal to a sinusoidal diameter (23.5 µm [29]). As is illustrated in Figure 7, many (quasi-)two-dimensional lobules are needed to fill the same volume (and thus preserve mass balance) as single three-dimensional lobule. The difference between R_liv∶lob_ and the actual number of lobules indicates that 100 simulated lobules are currently needed to fill the space of a single physiologic lobule.

Using the lobule geometries (given in Figure 3) ensembles of ten lobules were used for simulating blood flow. For each geometry the flow was simulated for an oral exposure of 10 µMol total (equivalent to 0.03 mg per kg body weight for a 200 molecular weight compound and a 70 kg subject) with an intrinsic hepatic clearance due to metabolism of 10 µL/min/million hepatocytes. We compared the average concentration throughout the lobule 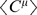, as predicted by our approach, with the prediction C_liv_ for a PBPK model with a well-mixed liver compartment with equivalent metabolic clearance 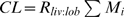 (*i.e. the* product of the clearance per hepatocyte, the total number of hepatocytes in a lobule, and the effective number of lobules R_liv∶lob_). It is important to note that the overall pharmacokinetics depends on the lobule layout because the effective number of lobules R_liv∶lob_ is determined by volume alone and therefore the total clearance of the liver depends on the number of hepatocytes relative to the volume of the lobule.

**Figure 8:**
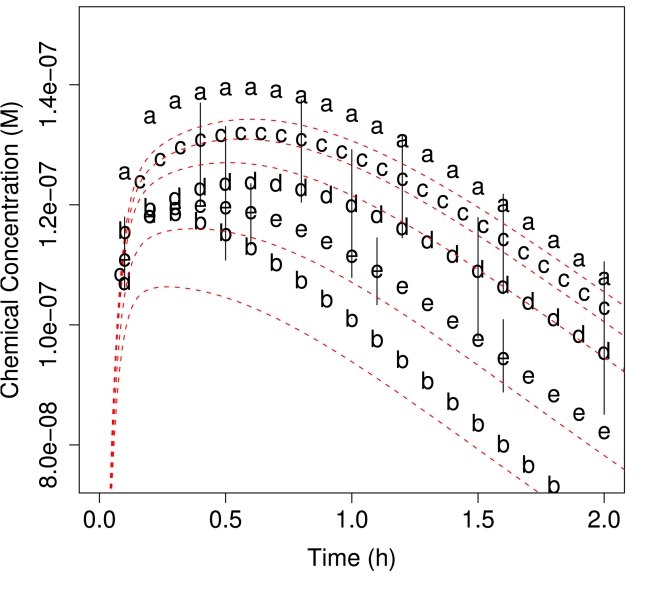
Average concentration throughout lobule for the five morphologies depicted in Figure 3. The ensemble average for all five lobules is very similar to the well-mixed lobule prediction (indicated by the dashed line) however the different morphologies produce different whole-liver clearances because the number of hepatocytes as a fraction of the volume of the simulated lobule is geometry-dependent.

Though the overall clearance varied with geometry, the impact of different geometries on the average concentration in the lobule was small. As shown in Figure 8, for the assumed metabolism rate the mean predicted concentration did not vary greatly from what would be predicted for a more traditional well-mixed compartment. To compare results between geometries the concentrations were scaled by C_liv_ predicted for the appropriate CL. We find that in all cases the predicted average concentration slightly exceeds the well-mixed PBPK prediction, but that otherwise the pharmacokinetics are very similar.

Plotted on the right-hand side of Figure 3 is the radial-dependence of concentration on position relative to the central vein at t_max_ – the time at which the lobule reaches maximum average concentration, 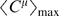. In all cases the mean concentration decreased slightly from the portal triads to the central vein – the predicted concentration was similar to the parallel tubes model. Thus, the mean predictions were similar to typical approaches for predicting liver concentrations.

Geometry had a much greater impact on the variability in predicted concentrations Figure 3. For all the lobules with random branching great variability was observed at the edges of the lobule, maximally distant from the central vein. Some regions receive slightly higher concentrations while other, stagnant regions received almost none. This supports the idea of considering sinusoidal topology for estimating changes in the local environment of a hepatocyte in addition to radial location between the central vein and the portal triad (*i.e.* zone I, II, or III). Since there were not large differences between the predictions for the three lobules with random branching, we arbitrarily chose to simulate lobules with six portal triads and 10% chance of branching (geometry c in Figure 3) for the remained of the studies in this paper. A larger ensemble of fifty lobules was generated for these studies.

### Chemical Kinetics

**Figure 9:**
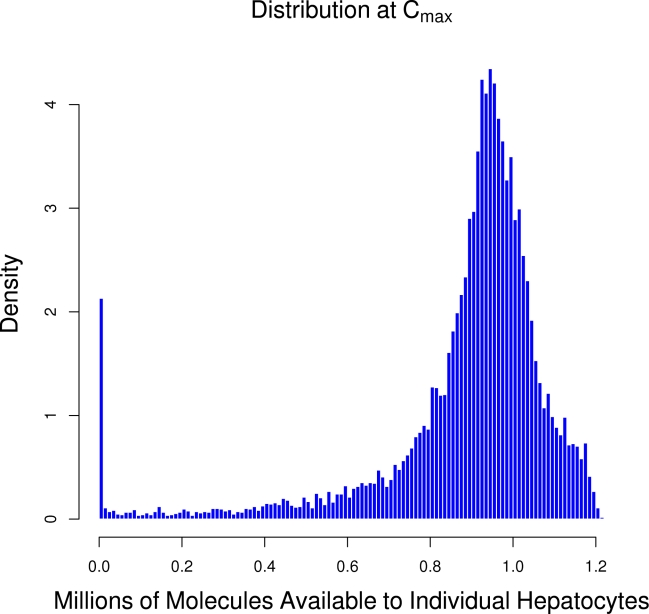
The distribution of the number of molecules at each hepatocyte following a total dose of 10 µMol.

To test whether a continuum approximation (ODEs) was appropriate for modeling mass transfer in the sinusoidal graph we estimated the number of molecules at a hepatocyte. If the number of molecules at higher concentrations is not large enough a stochastic approach [33] would be preferable. As shown in Figure 9, the upper bound on the number molecules at a total dose of 10 µM is was nearly a million molecules per hepatocyte, as calculated by multiplying the concentration in the sinusoid adjacent to each hepatocyte and dividing by the number of hepatocytes accessing that sinusoid. Though a small fraction of hepatocytes are exposed to almost no molecules, a continuum approach appears appropriate.

**Figure 10:**
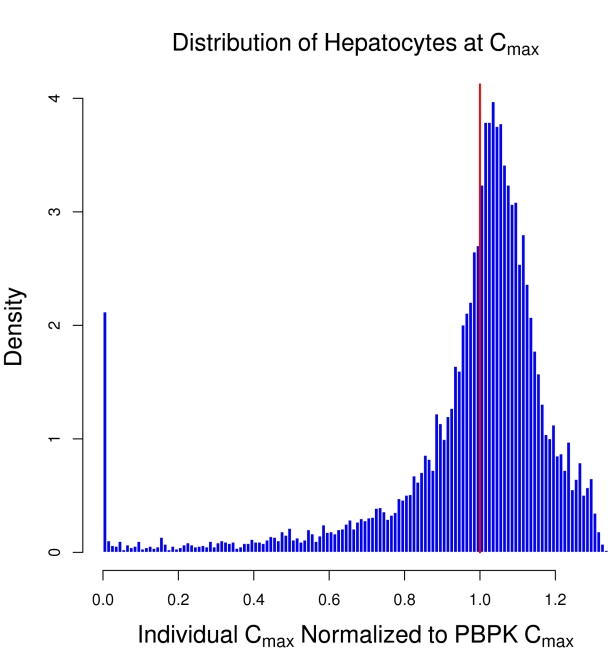
The distribution of maximum concentration experienced by hepatocytes relative to the prediction of a well-mixed PBPK model (solid line).

The maximum concentration in the tissue following a dose is a commonly used measure of tissue exposure in pharmacokinetics. For the simulated lobule a local C^μ^_i,max_ can be calculated for each hepatocyte as a result of different sinusoids receiving different concentrations. Figure 10 shows the distribution of C^μ^_i,max_ experienced by all the hepatocytes in an ensemble of fifty lobules with intrinsic hepatic metabolic clearance of 10 µL/min/million hepatocytes. The values have been normalized to the C_max_ predicted for a well-mixed liver with the same overall metabolic clearance (indicated but the solid line). The peak for the distribution is in excess of the well-mixed prediction, while the breadth is quite wide, indicating that at this rate of metabolism some hepatocytes receive exposures nearly 40% greater than would be predicted for a well-mixed liver while others receive almost no exposure.

**Figure 11:**
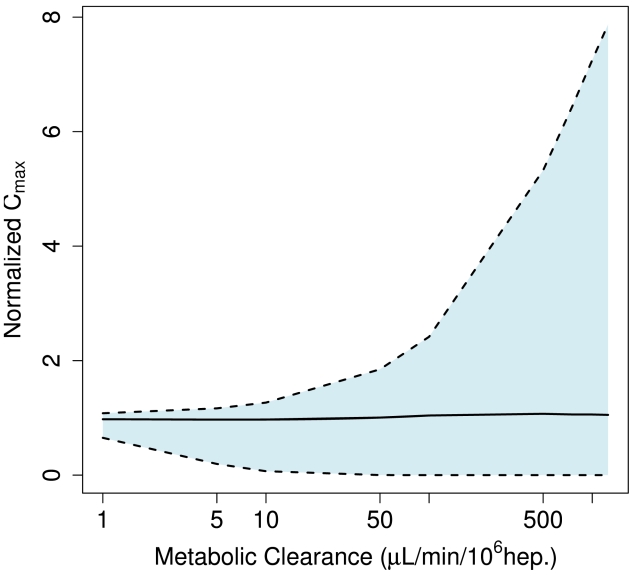
The breadth of the distribution of maximum exposure received by individual hepatocytes, *i.e.* variability in exposure, grows with the clearance rate. The shaded region indicates the 95% interval.

Ito and Houston [15] summarize a range of intrinsic metabolism rates including values as low as 1.4 µL/min/million hepatocytes (caffeine) and as large as 1800 µL/min/million hepatocytes (propranolol). This wide variability in metabolism rate has consequences for the variability predicted across the lobule. As shown in Figure 11, the variability in exposure received by different hepatocytes grows from a few percent to nearly 800% for a metabolism rate of 1000 µL/min/million hepatocytes. For rapid metabolism those hepatocytes first exposed to blood from the portal triad receive eight times the exposure that would be predicted for a well-mixed liver, while downstream hepatocytes receive almost no exposure to the parent compound.

Heterogeneity within the lobule is dynamic [34]; a low metabolism rate may be due to limited distribution of metabolizing enzymes, while a high rate of metabolism may lead to induction of enzymes, perhaps heterogeneously. Both of the distributions in Figure 9 and Figure 10 are broad, indicating that the average response of the ensemble is not necessarily characteristic of the response of any one simulated lobule. Given that these and other variability have been observed, any model of hepatic effect that depends upon local concentrations, particularly threshold models, may have a different response for a spatially-extended simulation than with a well-mixed simulation. The relevance of this heterogeneity will depend on the parameter regime – for low metabolism and little variability, the well-mixed approximation is likely to be sufficient. If large variability is present, *e.g.* for rapidly metabolized compounds, it may be crucial to determine which hepatocytes receive large exposures. This is especially useful for modeling spatial effects such as the development of lesions in one region, but not another.

### Impact of Microdosimetry on Hepatocellular Responses

We conducted a preliminary analysis of the cellular effects due to microdosimetry using a simple agent-based model for hepatocytes. Each agent was defined by a fixed, identical xenobiotic metabolism rate, and functional states that were updated at each time step via state transition rules. A simple approach was used to encode probabilistic state transition rules conditioned on inputs from the agent environment. Future cellular models will be able to take better advantage of the freedom to proliferate and move provided by this approach since flow for a new arrangement can be determined rapidly by updating the sinusoid and contact graphs. Here we considered normal hepatocytes and cell death following exposure to threshold cytotoxic concentration. The ABM was integrated with the sinusoidal flow model with each being updated alternately. We simulated twelve minutes of the flow followed by eight iterations of the ABM – intended to be sufficiently small time periods for each model to respond realistically to changes in the other. Experimental verification will be needed to determine the appropriate time scales.

Given the current cellular model and the predicted increase in variability with metabolism rate shown in Figure 11, two types of comparisons were made: a spatially-extended hepatic lobule with an approximate “parallel tube” model (given by the lobule geometry in Figure 3a) and variability due to rapid metabolism for low (1 µL/min/million hepatocytes) and high (1000 µL/min/million hepatocytes) rates of metabolism. An arbitrary threshold of chemical concentration has been assumed, above which cell stress and apoptosis become much more common. Since different metabolic clearances and lobule geometries lead to different pharmacokinetics the simulations were normalized by varying the threshold for enhanced apoptosis – the threshold was set to 110% of the maximum average lobule concentration predicted for each configuration.

**Figure 12:**
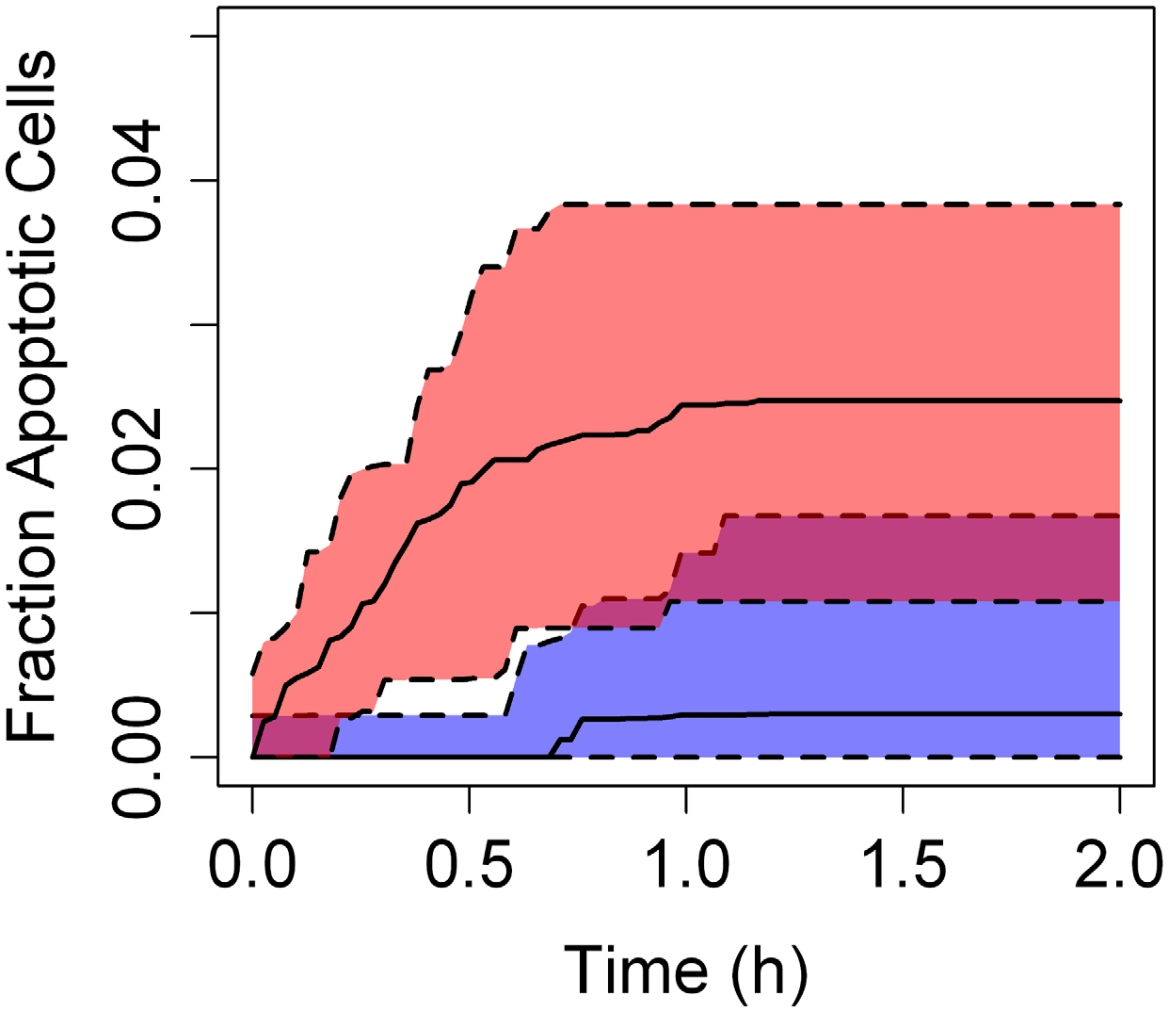
The predicted number of apoptotic cells, caused by locally exceeding a threshold of 110% of the maximum average liver concentration, is negligible for a spatially-extended lobule when the metabolism rate is low (lower curve). For a rapidly metabolized compound (upper curve) variability in exposure causes some apoptosis in the spatially-extended lobule. The shaded region indicates the 95% interval.

For a well-mixed lobule, a threshold in excess of maximum lobule concentration should have no effect. Instead, as shown in Figure 12 we observed that spatial heterogeneity in toxicant concentration across the lobule enhanced cell injury before the chemical was cleared. This effect was not observed in the approximate parallel tubes model. Enhanced cell death was not observed at low xenobiotic metabolism rates in the spatially-extended lobule. Though there is some baseline apoptosis at the lower metabolism rate, there is roughly five times greater apoptosis for higher metabolism, i.e. greater variability in exposure. This suggests that lobular geometry is not solely responsible for the cell behavior and hepatocyte metabolism is required for the variability in the cellular response. Variation in cellular responses is frequently observed [17] and is thus physiologically relevant. While additional work is required to evaluate the responses in our model, these findings suggest the value of spatially extended tissue level models of microcirculation and cellular dynamics.

## Discussion

We have described a microdosimetry model to relate environmental exposures to cellular exposures. This is only a step toward developing virtual tissues that can predict the *in vivo* consequences of chemical exposure based upon *in vitro* information.

The liver lobule is known to be spatially heterogeneous [18], [34]. Zonal differences between central and peripheral hepatocytes include oxygen availability, hormone concentration, expression of metabolizing enzymes, (e.g., CYP 3A4), gluconeogenesis, and glycolysis [18]. One clear conclusion of this modeling work is that morphology of the liver alone is insufficient to explain the observed zonation in hepatocyte function or even gradients in concentration across the lobule. We observed variations that are driven by the action of hepatocytes, *i.e.* metabolism, and not by geometry alone.

A model for a spatially-extended hepatic lobule sets the stage for investigating emergent behavior in models of hepatocyte function. If the action of hepatocytes creates spatial variation across the lobule then any cellular dynamic response that depends on chemical or nutrient concentration may in turn be altered, which could be a prelude to zonal patterns of biological functions. More extreme effects, such as central lobular necrosis, may be due to the transformation of the compound via metabolism into a more potent compound or zone-dependent variation in sensitivity of the hepatocytes.

In contrast to the well-stirred model of the liver, the simulated lobule provides a means of accessing a variety of inter- and intracellular dynamics. Though the results we obtain are in some respects similar to previous models, we gain the additional capability of allowing hepatocyte-specific dosimetry as well as the potential to alter lobule geometry, *e.g.* lesions or necrosis, in response to chemical injury. Since numerical approaches often allow even large systems of ODEs to be solved much more rapidly than analogous systems of PDEs [35] and since numerous algorithms exist for analysis of graphs [36], we believe this approach is tractable for simulating sub-chronic and chronic xenobiotic exposure scenarios while preserving mass-balance. Because we use a flexible graphical model of tissues, the remaining micro-anatomic structures (other cell types, extracellular matrix, bile ducts, etc.) can be included incrementally without significant changes in our approach.

In contrast to computationally intensive, spatially continuous approaches such as fluid dynamics, this graph-theoretic approach has hopefully sacrificed little physiologic detail but gained a great deal in terms of computational efficiency. Calculating hemodynamical flow on a graph allows rapid determination of flow given minimal boundary conditions, which will be especially useful for recalculating flow as morphology changes (e.g. lesion formation) or as individual sinusoids are temporarily blocked (e.g. Kupffer cells). A faster dosimetry model allows the focus to center on cellular phenotypes, which are the key to modeling disease pathogenesis. A computationally-tractable approach allows for simulating the long run times associated with sub- and chronic toxicity studies as well as simulating large populations.

We evaluated our approach to hepatic blood flow in three ways. First, we qualitatively tuned the appearance of the lobule to match actual physiology. Second, we compared the predicted pharmacokinetics for our spatially-extended lobule with traditional approaches, finding regimes in which our approach reduced to the well-mixed liver and the parallel tubes model. Third, we quantitatively compared the flow predicted for a rat with observations made *in vivo* of actual flow. Though all three lines of evaluations supported our approach, they also all pointed toward further refinements that may be necessary for simulating dose-response.

This work addresses the dose portion of the dose-response curve, allowing assessment of how changes in exposure impact the hepatic lobule. The greater body of work remains with modeling response. Sufficiently complex models for hepatocellular dynamics, and eventually models for additional cell types, especially the Kupffer cells responsible for inflammatory responses, must be developed before we arrive at a useful model for homeostatic liver function. It remains to be seen whether three-dimensionality or even a departure from the classical lobule paradigm to simulate multiple lobules will be needed.

To establish the safety of a compound one ideally finds the dose-response curve for various toxicity endpoints, so that an acceptable level of exposure can be determined. Currently the gold standard of toxicology is animal testing, but the need and desire for i*n vitro* testing is growing. An *in silico* model for predicting dose-response would, at a minimum, provide a screen for prioritizing compounds that requiring further testing and perhaps may ultimately be able to predict *in vivo* consequences for the large number of compounds for which there is little or no toxicity data.

The multiscale approach describe here is intended to be fast and verifiable, and would allow the determination of whether an observed *in vitro* response is relevant *in vivo*. The limitations in developing a homeostatic model of liver function are not computational, but biological. Additional data is needed, especially information on the statistical distribution of lobule morphology and the determination of cell state in response to local inputs. This model provides a framework for making use of two types of readily available data – histopathology slides and *in vitro* measures of cell function. In all likelihood direct comparison to liver toxicology data will be met initially with more failures than successes, but where we initially fail we will learn.

Histopathology images have long been used to obtain information on microanatomic regions, vasculature, individual cells, cell types, and cell phenotypes from two- and three-dimensional images. Though traditionally time-intensive, advances in automated extraction of information from histopathology images are making it possible to analyze these images at a single cell resolution [37], [38], [39]. Additionally it is possible to extract information about the functional state of cells using cytomorphologic features or molecular markers [40]. Though cell-scale assay technology is still developing, it will be essential for fully calibrating and evaluating models such as this in order to provide simulated *in vivo* context for the results of *in vitro* assays.

True variability in the response of a given hepatocyte is either a product of independent microdosimetry and cell variability, or is a function of the two, depending on the degree of correlation. To determine the significance of a chemical perturbation it is not enough to understand the cellular dynamics, but also the context in which those dynamics exist – i.e., microdosimetry.

## Methods

### Microdosimetry Model of a Lobule

We have implemented a microdosimetry model for relating whole-body chemical exposures to cell-scale concentrations. The model is written in the freely available statistical language R, version 2.8.1 [41].

### Generating Sinusoidal Morphology

Given morphologic parameters N_t_, the number of portal triads; N_s_, the number of sinusoids per source/sink; P_branch_, the probability of a sinusoid branching; and D_max_, the size of the lobule, and calculating θ_CV_ is the angle to the central vein, given current position:

1. Place central vein
2. For each of N_s_ sinusoids:
  1. Select initial angle θ^0^_s_
  2. Place sinusoidal primitive on edge of central vein at θ^0^_s_
  3. Call the sinusoid placement algorithm (SPA) with θ_s_ = θ^0^_s_
  4. Increment θ^0^_s_ approximately 2π/N_s_
3. For each of N_t_ portal triads:
  1. Select initial angle θ_t_
  2. Place a periportal vein at angle θ_t_ and distance 0.8*D_max_
  3. For each of N_s_ sinusoids:
    1. Select initial angle θ^0^_s_ = θ_CV_
    2. Place a sinusoid primitive on edge of the periportal vein at θ^0^_s_
    3. Call SPA with θ_s_ = θ^0^_s_
    4. Increment θ^0^_s_ approximately 2π/N_s_
  4. Place an arteriole randomly at the edge of the periportal vein
  5. For each of N_s_ sinusoids:
    1. Select initial angle θ^0^_s_ = θ_CV_
    2. A sinusoid primitive is placed at the edge of the arteriole at θ^0^_s_ and the SPA is called with θ_s_ = θ^0^_s_
    3. θ^0^_s_ is incremented approximately 2π/N_s_

**Recursive Sinusoid Placement Algorithm (SPA):**

1. Calculate the potential position of the next sinusoidal primitive using θ_s_
2. If either the distance from the central vein exceeds D_max_ or the potential location overlaps with a previously placed sinusoid, then
  1. Return
3. If a randomly drawn number [0,1] is less than P_branch_, then
  1. Randomly select θ'_s_ from the interval [θ_CV_−π/2, θ_CV_+π/2]
  2. Call SPA with angle θ_s_ = θ'_s_
4. θ_s_ is randomly perturbed
5. Call SPA

### Reducing the Complexity of the Sinusoidal Graph

The aggregation process is performed using the following algorithm:

1. All sinusoid primitive nodes are assigned a corresponding aggregate node (CAN) initially set to NULL
2. For each sinusoid node I adjacent to the central vein, if the CAN is NULL, then,
  - if the number of sinusoid neighbors N^i^_n_ = 1, a “dead end” CAN is created,
  - if N^i^_n_ = 2, a “straight” CAN is created
  - if N^i^_n_>2 a “branch” CAN is created
  - For each neighbor j, If N^i^_n_ = N^j^_n_ then,
    - The CAN for j is set to the CAN for i unless the CAN is a straight node already consisting of 5 sinusoid primitive nodes
    - Step 2d is called recursively for node j
3. Repeat step 2 for each arterial and venous source
4. For each branch CAN i,
  - For each neighbor j if j is also a branch then CAN i is absorbed into CAN j
5. For each straight CAN i, if there is now only one neighbor and that neighbor is a branch CAN, merge i with the neighbor
6. For each branch CAN i, if there is only one neighbor convert I into a dead end CAN
